# The epidemiology of personality disorders in Norway: a nationwide patient registry study

**DOI:** 10.1007/s00127-026-03083-7

**Published:** 2026-03-24

**Authors:** Mathias Valstad, Nikolai Haahjem Eftedal, Tianfang Yang, Elfrida Hartveit Kvarstein, Ted Reichborn-Kjennerud, Eivind Ystrom, Nikolai Czajkowski

**Affiliations:** 1 PROMENTA Research Centre, Department of Psychology, University of Oslo, Oslo, Norway; 2 Department of Research and Innovation, Division of Mental Health and Addiction, Oslo University Hospital, Oslo, Norway; 3 Institute of Clinical Medicine, University of Oslo, Oslo, Norway; 4 PsychGen Centre for Genetic Epidemiology and Mental Health, Norwegian Institute of Public Health, Oslo, Norway; 5 Centre for Research on Equality in Education, Faculty of Educational Sciences, University of Oslo, Oslo, Norway

**Keywords:** Personality disorders, Borderline, Specialist health care services, Incidence rates, Time trends

## Abstract

**Purpose:**

To better understand the developmental course of personality disorders and the scope of diagnosis and treatment in specialist health care, we estimate incidence rates of specialist health care personality disorder diagnosis by age, gender, and calendar time, as well as lifetime risks, in the whole population of Norway. The measures provided are foundational for developmental theories of personality disorders, for treatment planning, and for later epidemiological studies.

**Methods:**

We studied every person born 1940 or later and resident and at risk in Norway between January 1st, 2010 and December 31st, 2022 (*n* = 6,040,689). We used Poisson regression with penalized cubic splines to model incidences of personality disorder diagnosis across age, gender, and calendar time, and calculated lifetime risks while accounting for competing risks of death and emigration.

**Results:**

Across specific disorders, incidence was highest in young adulthood. The lifetime risk of any personality disorder diagnosis in specialist health care was 5.4%. The two most incident specific diagnoses were borderline and avoidant personality disorders. Generally, incidence increased through the study period, with an incidence rate ratio (IRR) of 1.21 between 2022 and 2010 – this trend was largely due to borderline (IRR = 1.40) and avoidant (IRR = 1.66) personality disorder becoming more common. Lifetime risk of any personality disorder diagnosis was higher among women (cumulative incidence = 6.7%) than among men (cumulative incidence = 4.1%), and increased more through the study period among women (IRR = 1.34) than among men (IRR = 1.03).

**Conclusion:**

Personality disorders are common in the Norwegian population, often first identified in specialist health care during early adulthood.

**Supplementary Information:**

The online version contains supplementary material available at https://doi.org/10.1007/s00127-026-03083-7.

## Introduction

Personality disorders are characterized by prevailing impairments in emotional and interpersonal functioning, and by associated adverse behaviors. They can drastically reduce the patient’s quality of life [1], economic prospects [2], and life expectancy [3, 4]. Information on how personality disorders distribute across age, calendar time, and gender is clinically and theoretically paramount, and can be ascertained through epidemiological means. The understanding of personality disorders within psychiatry has gone through and is still going through a series of developments [5, 6], including the discoveries of effective treatments like dialectical behavioral therapy [6–8] and advances in measurement and diagnosis with standardized procedures involving structured clinical interviews [9]. These developments permit the examination of personality disorder in the full population using medical registry data, and at the same time provide relevant historical context for interpreting findings from this data. A medical registry approach is especially pertinent in countries with universal health care and nationwide registry coverage, like Norway. In this context, it provides complementary information to diagnostic interview studies in samples from the population [10–12] and comes with the advantages of negligible selection bias and a strong ecological validity with its close mirroring of actually existing clinical practices [13, 14].

Medical registry based studies of personality disorders in entire populations over time are still rare. One nationwide study from New Zealand found an increase in the number of people diagnosed with personality disorders from 2009 to 2016, with most diagnoses being either borderline or dissocial personality disorder [14]. This study did not focus on the estimation of incidence rates or risks. In contrast, another study following the full Danish population between 2000 and 2012 estimated lifetime risks for any personality disorder diagnosis in specialist health care at 3.45% for males and 6.66% for females [15]. In the same population, between 2004 and 2021, diagnostic incidence was slightly increased for females (7.13%) but slightly decreased for males (3.09%) [16]. These studies did not formally model change in diagnostic incidence over calendar time. Further, since these Danish studies had a broad scope, examining incidences and lifetime risks across the spectrum of mental disorders, they did not focus on specific personality disorder diagnoses other than borderline and dissocial [15, 16].

Here, we analyse the incidence rates over time (age and calendar years), lifetime risk, and incidence rate ratios between genders and between calendar years in the Norwegian population for all 11 ICD-10 personality disorder diagnoses. We ask the questions: (i) when in life do people receive a personality disorder diagnosis, (ii) what is the lifetime risk of being diagnosed with a personality disorder, (iii) how has personality disorder incidence changed over the study period, and (iv) to what extent do the above trends differ between genders.

## Methods

### Study sample

In this nationwide, population-based retrospective open cohort study, we linked the Norwegian Population Registry, the Norwegian Patient Registry [17], and the Control and Payment of Health Reimbursements Database (KUHR), and studied every person born 1940 or later and resident and at risk in Norway between January 1st, 2010 and December 31st, 2022. Individuals were included to follow-up at birth, immigration to Norway, or January 1st, 2010, whichever appeared last, and were censored at personality disorder diagnosis, emigration, death, or December 31st, 2022, whichever appeared first. Prevalent cases at January 1st, 2010, as identified by diagnosis in KUHR or the Norwegian Patient Registry during a washout period (January 1st, 2006–December 31st, 2009), were not included to follow-up.

### Diagnosis of personality disorder in Norwegian specialist health care

Norwegian specialist health care follows the International Classification of Diseases (ICD)-10 diagnostic manual, which differentiates between categorically separate personality disorders. These include paranoid (F60.0), schizoid (F60.1), dissocial (F60.2), emotionally unstable or borderline (F60.3), histrionic (F60.4), anankastic (F60.5), avoidant (F60.6), and dependent (F60.7) personality disorder, along with other specific (F60.8), unspecified (F60.9), and mixed (F61) personality disorder. In the Norwegian specialist health care, each contact with a patient is registered with a code, most of which indicate a diagnosis. Hospitals are required to submit all registrations to the Norwegian Patient Registry. The Norwegian Patient Registry therefore contains complete data on personality disorder diagnosis in specialist health care, including the psychiatric and somatic health care areas, outpatient clinics and hospitals, child and adult health care, and public and private clinics.

### Analytic approach

For each personality disorder F60.x-F61 and for any personality disorder, we count total person-years of follow-up (Y) as well as the number of incident cases (D) per calendar year and age (also split by gender for the gender-specific analyses). We then fit a series of Poisson regression models on incident cases over total person-years using penalized cubic splines, and predictors varying between models, following an approach previously employed in Danish epidemiological studies [16, 18]. In model 1A, D/Y is expressed as a function of age, using penalized cubic splines with a maximum of 15 knots. In model 2, D/Y is expressed as a function of age and calendar year, using penalized cubic splines with a maximum of 15 knots for age and 3 knots for calendar year. In model 3A, D/Y is expressed as a function of age, using penalized cubic splines with a maxium of 15 knots and gender, which is just categorical, and the interaction between age and gender. In model 4, D/Y is expressed as a function of age as usual, calendar year as in model 2, gender as in model 3, interactions between age and gender as in model 3, and lastly of the interaction between calendar year and gender. These models of incidence rates were fitted using the gam() function in the ‘mgcv’ package for R [19].

In the presence of competing risks like emigration and death, there is no longer a one-to-one relation between incidence rates and risks [20]. However, risks can be calculated as cumulative incidences while taking the competing risks of emigration and death into account by scaling the failure integral proportionally to people surviving at each continuous age, where survival is defined as not receiving the diagnosis of interest, not emigrating, and not dying [20]. With this procedure, model 1B estimates the disease-specific cumulative incidences regardless of gender, while model 3B estimates this separately in each gender. We define lifetime risk as the disorder-specific cumulative incidence at age 82 (the average life expectancy in Norway). These models of cumulative incidences in the presence of competing risks were fitted using the prodlim() function in the ‘prodlim’ package for R [21].

For each diagnosis, we report 9 relevant statistics: peak incidence (from model 1A), cumulative incidence across the expected lifespan (ages 0–82; from model 1B), incidence rate ratio (IRR) between 2022 and 2010 (from model 2), peak incidence from each of the genders (from model 3A), cumulative incidence across the lifespan (ages 0–82) for each of the genders (from model 3B), and incidence rate ratio between 2022 and 2010 for each of the genders (from model 4).

### Sensitivity analyses

Since we do not have diagnostic data from other countries than Norway, false incident cases might be higher among immigrants than in people born in the country. To address this issue, we compare the main lifetime risk estimates to estimates from people born in Norway only. Further, since the washout period used for identification of prevalent cases at study onset is limited, there might be false incident cases, especially among older adults, if someone was diagnosed with a personality disorder prior to but not during this limited period. To address this issue, we perform sensitivity analyses with the washout period extended in time up until December 31st, 2021.

## Results

We followed 6,040,689 Norwegian residents through the years 2010 to 2022, amounting to a follow-up of at least 63,460,438 person-years, depending on the specific diagnosis (higher follow-up time for less incident personality disorders). Throughout the study period, there were 61,077 incident cases of personality disorder. The lifetime risk, calculated as the cumulative incidence from 0 to 82 years of age, of any ICD-10 personality disorder diagnosis in Norwegian specialist health care was 5.4% (95% CI: (5.3%, 5.4%)). The most frequent personality disorders were avoidant (cumulative incidence = 1.8%, 95% CI: (1.8%, 1.9%)) and borderline (cumulative incidence = 1.8%, 95% CI: (1.7%, 1.8%)) personality disorder (Table 1). The lifetime risk of personality disorder diagnosis in specialist health care differed between genders, being higher in women (cumulative incidence = 6.7%, 95% CI: (6.6%, 6.8%)) than in men (cumulative incidence = 4.1%, 95% CI: (4.1%, 4.2%)).

**Fig. 1:**
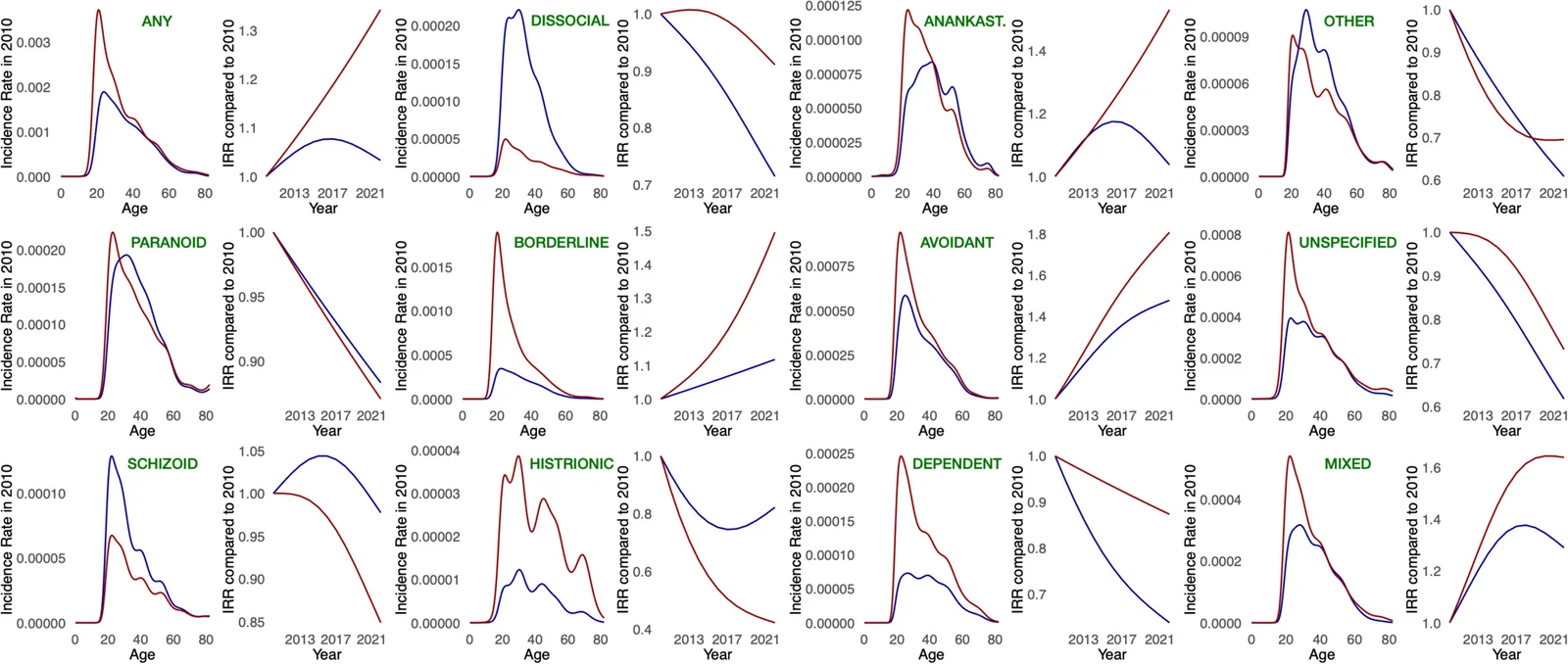
Incidence rates by age, gender (red = female, blue = male), and calendar year for any personality disorder and all the specific personality disorders F60.x and F61. Note that there are large differences between the y axis ranges, reflecting differences in incidence between diagnoses. The models depicted (Model 4) allow interactions between age and gender, and calendar year and gender, but not between age and calendar year. To get a calendar year’s age curve, the 2010 curve (left) is streched by that year’s incidence rate ratio (right)

Incidence rates across life for any personality disorder, and for all the specific ICD-10 personality disorder diagnoses, are shown in Fig. 1. These show incidences beginning in adolescence and increasing quickly in late adolescence/early adulthood, followed by declines toward zero incidence through adulthood at rates specific to each categorical diagnosis. To illustrate, the risk of being diagnosed with any personality disorder before turning 18 is 0.1% (95% CI: (0.1%, 0.1%)).

Through the study period, 2010–2022, incidences for any personality disorder increased (IRR = 1.21, 95% CI: (1.18, 1.24)). These increases primarily reflect trends for the two most incident diagnoses: avoidant (IRR = 1.66, 95% CI: (1.59, 1.74)) and borderline (IRR = 1.40, 95% CI: (1.33, 1.46)) personality disorders. Of the other specific diagnoses, only anankastic personality disorder increased in incidence over the study period (IRR = 1.29, 95% CI: (1.15, 1.44)), all the others became less used in specialist health care. The two nonspecific personality disorder diagnoses, unspecified (F60.9) and mixed (F61), switched places, with the former decreasing (IRR = 0.68, 95% CI: (0.64, 0.72)) and the latter increasing (IRR = 1.48, 95% CI: (1.40, 1.57)) in incidence (Table 2).

**Table 1:** Cumulative incidences at age 82 are calculated while taking the competing risks of death and emigration into account

Diagnosis	Estimated lifetime risk in % (with 95% CI)			Age of peak incidence		
	Women	Men	All	Women	Men	All
Paranoid	0.44 (0.42, 0.45)	0.43 (0.42, 0.45)	0.43 (0.42, 0.45)	22.8	31.2	24.1
Schizoid	0.13 (0.12, 0.14)	0.23 (0.22, 0.25)	0.18 (0.18, 0.19)	22.2	22.1	22.2
Dissocial	0.09 (0.08, 0.09)	0.39 (0.37, 0.41)	0.24 (0.23, 0.25)	21.8	29.9	23.1
Borderline	2.85 (2.80, 2.89)	0.73 (0.71, 0.76)	1.77 (1.74, 1.79)	20.2	22.3	20.4
Histrionic	0.06 (0.06, 0.07)	0.02 (0.02, 0.03)	0.04 (0.04, 0.05)	29.7	30.2	29.8
Anankastic	0.34 (0.32, 0.35)	0.24 (0.23, 0.26)	0.29 (0.28, 0.30)	23.4	38.6	30.8
Avoidant	2.27 (2.23, 2.31)	1.42 (1.39, 1.45)	1.84 (1.81, 1.86)	22.0	25.0	23.0
Dependent	0.45 (0.44, 0.47)	0.16 (0.15, 0.17)	0.30 (0.29, 0.31)	22.4	26.4	22.8
Other specific	0.16 (0.15, 0.17)	0.19 (0.18, 0.20)	0.18 (0.17, 0.18)	20.8	29.0	28.1
Unspecified	1.27 (1.24, 1.30)	0.80 (0.78, 0.82)	1.03 (1.01, 1.05)	21.4	22.6	21.7
Mixed	1.44 (1.41, 1.47)	0.93 (0.90, 0.95)	1.18 (1.16, 1.20)	22.2	28.0	23.0
Any PD	6.71 (6.64, 6.78)	4.11 (4.06, 4.16)	5.38 (5.34, 5.43)	20.8	23.8	21.6

Non-overlapping confidence intervals between genders, apparent f or all diagnoses except paranoid personality disorder, are indicative of a gender difference in risk. CI: confidence interval. Age of peak incidence is the age at which the diagnosis is most incident, i.e. the maximum of the incidence curve in Fig. 1

Two diagnoses had higher lifetime risk in men than in women: schizoid (cumulative incidence = 0.2% in men vs. cumulative incidence = 0.1% in women) and dissocial (cumulative incidence = 0.4% in men vs. cumulative incidence 0.1% in women). Otherwise, risk was higher in women or there was no difference between the genders (Table 1). Overall, gendered diagnosis was exacerbated through the study period. For any personality disorder, more incident in women than in men at study onset, incidence rate ratios between 2022 and 2010 were also substantially higher in women (IRR = 1.34, 95% CI: (1.30, 1.39)) than in men (IRR = 1.03, 95% CI: (0.99, 1.08)). The same pattern was observed in the two most incident personality disorder diagnoses. 2022 vs. 2010 incidence rate ratios were higher in women (IRR = 1.50, 95% CI: (1.42, 1.57)) than men (IRR = 1.12, 95% CI: (1.01, 1.23)) for borderline and in women (IRR = 1.81, 95% CI: (1.71, 1.92)) than men (IRR = 1.48, 95% CI: (1.38, 1.59)) for avoidant personality disorder.

**Table 2:** Increase in incidence from 2010 to 2022 with confidence intervals

Diagnosis	Incidence rate ratios, 2022 over 2010 (with 95% CI)		
	Women	Men	All
Paranoid	0.87 (0.76, 0.99)	0.88 (0.78, 1.00)	0.88 (0.80, 0.96)
Schizoid	0.85 (0.67, 1.08)	0.98 (0.82, 1.16)	0.93 (0.81, 0.97)
Dissocial	0.91 (0.68, 1.22)	0.71 (0.62, 0.82)	0.75 (0.66, 0.84)
Borderline	1.50 (1.42, 1.57)	1.12 (1.01, 1.23)	1.40 (1.33, 1.46)
Histrionic	0.42 (0.30, 0.59)	0.82 (0.49, 1.39)	0.51 (0.39, 0.68)
Anankastic	1.53 (1.32, 1.77)	1.04 (0.88, 1.23)	1.29 (1.15, 1.44)
Avoidant	1.81 (1.71, 1.92)	1.48 (1.38, 1.59)	1.66 (1.59, 1.74)
Dependent	0.87 (0.77, 0.99)	0.64 (0.52, 0.78)	0.80 (0.72, 0.89)
Other specific	0.69 (0.56, 0.86)	0.61 (0.50, 0.73)	0.64 (0.56, 0.74)
Unspecified	0.73 (0.68, 0.79)	0.62 (0.56, 0.68)	0.68 (0.64, 0.72)
Mixed	1.64 (1.52, 1.76)	1.29 (1.18, 1.41)	1.48 (1.40, 1.57)
Any PD	1.34 (1.30, 1.39)	1.03 (0.99, 1.08)	1.21 (1.18, 1.24)

A confidence interval not including 1 indicates a significant change in incidence from 2010 to 2022. Non-overlapping confidence intervals between the genders indicates that the change from 2010 to 2022 is different between men and women

We performed two sets of sensitivity tests, recounted in Supplementary Table 1. First, to assess the impact of false incidences from immigrants previously diagnosed abroad, we ran analyses with only individuals born in Norway. These analyses suggest that, if anything, the inclusion of immigrants in the main analyses lead to under- rather than overestimation of risk. One of the reasons for this is that the competing risk of emigration is higher among people not born in Norway (Supplementary Fig. 1). Second, to assess the impact of false incidences from individuals diagnosed prior to but not within the washout period, we extended the washout period until December 31st, 2021. Again, this procedure and the standard procedure led to similar, though not identical, cumulative incidence estimates.

## Discussion

Using nationwide registry data, we show that personality disorder diagnoses are widely used in specialist health care, reflecting the prevalent nature of these mental health conditions. Age trends show an absence of diagnoses in childhood, a sudden rise in incidence in late adolescence with peaks in early adulthood, and declines in incidence through adulthood toward zero incidence in later life. Of note, personality disorder diagnosis became more incident in the Norwegian population from 2010 to 2022. Borderline and avoidant personality disorder were markedly more incident than the other specific diagnoses, and they also increased the most in incidence over the years of follow-up. Personality disorder diagnosis in general was more incident, and increased more in incidence, among women than among men.

The observed relative distribution of specific personality disorders differs markedly from estimates obtained using diagnostic interviews in community samples [11], even when sampled from a comparable population (Norway in the 1990s) [22]. For example, the incidence of borderline personality disorder stands out as higher than other diagnoses, while its prevalence in community samples does not [11, 22]. This underscores the complementary relationship between diagnostic interview studies and registry studies. Crucially, secondary health care diagnosis typically is the end point of a causal chain where functional impairment plays a substantial role [14]. Therefore, these differences between studies with different methodologies are likely to reflect differences in functional impairment between the personality disorder diagnoses. Further, some of these differences might be due to the pragmatic nature of clinical practice – rather than focusing solely on results from diagnostic interviews, clinicians often consider the utility of selecting one particular diagnosis over the other. Borderline personality disorder diagnosis could be assumed particularly useful by clinicians because it has become associated with relatively clearly defined treatment courses. Other diagnoses, like histrionic personality disorder, could be associated with stigma and therefore with less utility [23]. Finally, we note that avoidant personality disorder is more widespread, and dissocial personality disorder less widespread, in Norway than in other countries for which data is available [11, 14, 22].

On the other hand, our estimates of incidences and lifetime risks for personality disorders largely converge with estimates in studies using similar methodologies in comparable demographic contexts (Denmark) [15, 16]. The most recent of these studies [16] found lifetime risks for personality disorder diagnosis at 7.1% among women and 3.1% among men, comparable to 6.7% among women and 4.1% among men in the Norwegian population. This convergence suggests that Danish and Norwegian mental health professionals share a similar understanding of personality disorder diagnosis. Further, the lifetime risk of dissocial personality disorder diagnosis among women was 0.08% in Denmark and 0.09% in Norway, and among men it was 0.41% in Denmark and 0.39% in Norway. Further, the lifetime risk of borderline personality disorder was 2.95% in Danish and 2.85% in Norwegian women. The only divergence was between Danish and Norwegian men in borderline personality disorder risk, at 0.35% and 0.73%, respectively. The risks for specific personality disorders other than borderline and dissocial were not estimated in the Danish studies.

We find that the incidence of personality disorder diagnosis is low through childhood, though increasing from mid adolescence, peaking in early adulthood, then declining through later life. This pattern is common among mental illnesses. However, there is reason to believe that diagnostic practices shift this curve to the right. The ICD-10 diagnostic manual, still used by Norwegian clinicians through the period of study, explicitly states it is *“unlikely that the diagnosis of personality disorder will be appropriate before the age of 16 or 17 years”* [24]. In addition, clinicians might be reluctant to diagnose adolescents with personality disorder due to concerns about the stability of personality at that age and/or fear of stigma [5, 25–27]. Such practices contrast with the evidence that personality disorder, especially borderline personality disorder, is prevalent, measurable, and treatable in adolescents [5, 25, 28–30].

Personality disorder diagnosis is more common in women than in men, with the exceptions of dissocial, schizoid, and paranoid personality disorder. This pattern is found also in other populations [16]. The higher incidence of diagnosis in women is likely influenced by a combination of gender differences in health care-seeking behavior [31], clinician bias [32], and gender differences in the exposure to etiological factors [33].

The incidence of personality disorder diagnosis increased through the study period, though the increase was due entirely to a subset of specific diagnoses: borderline, avoidant, anankastic, and mixed personality disoder. The increase in diagnosis could in part reflect increases in the underlying symptomatology in the population. The period of follow-up coincides with a historical period where the Norwegian population appears to have become more burdened by general psychiatric symptoms [34]. This secular trend has been more pronounced in young women than in other demographics [34], potentially contributing to the gender differences we observe in the increase in personality disorder diagnosis. The increase in diagnosis could also in part reflect conscious efforts by Norwegian health authorities and researchers to standardize and improve diagnostic measurement. In 2012, a national advisory unit on personality disorders was established, aiming to educate health services and the public on personality disorders, diagnostic assessment, and management and understanding of lived experience (user) perspectives [35]. Further, the increase in diagnosis could also in part reflect more widespread administration of psychotherapeutic treatments for personality disorder or an increased awareness of their efficacy [6, 7]. For example, evidence-based structured treatments for borderline personality disorder with corresponding therapist training courses were increasingly available in Norway from 2010. Thus, disentangling the different factors contributing to increases in personality disorder diagnosis should be a focus of future research.

This study describes phenomena which are particular to Norway and which, though largely converging with epidemiological results from comparable countries (Denmark) [16], cannot immediately be generalized across national contexts. We also note that we study registered diagnoses, and that the results depend on the recording of diagnosis in Norwegian specialist health care. A further limitation is the potential for insufficient censoring of prevalent cases at the start of follow-up, since our washout period lasts only 4 years. By extending the washout period, we show that central epidemiological parameter estimates do not change in a manner consistent with considerable bias, but we cannot completely eliminate the potential for false incidences due to left truncation.

## Conclusion

Medical registry based studies in whole populations over time can increase the understanding of psychiatric disorders and of clinical practice. We show that personality disorder is common in Norway and that personality disorder diagnosis is widely used in Norwegian specialist health care. Diagnostic incidence peaks in early adulthood. The risk of being diagnosed with any personality disorder is higher in women than in men. The risk of receiving any personality disorder diagnosis in specialist health care increased through the study period, 2010–2022.

## Supplementary Information

Below is the link to the electronic supplementary material.


Supplementary Material 1


## Data Availability

Data sources are detailed in the manuscript.
