# Supplementary material for: The epidemiology of personality disorders in Norway: a nationwide patient registry study
Source: Soc Psychiatry Psychiatr Epidemiol. 2026 Mar 24;61(8):1629–36. doi: 10.1007/s00127-026-03083-7 (PMC13522045; doi:10.1007/s00127-026-03083-7)
Supplement: Supplementary file 1 [file 127_2026_3083_MOESM1_ESM.docx]

**Supplementary information**

*Supplementary table 1*

|  | **Standard** | | **Long washout** | | **No immigrants** | |
| --- | --- | --- | --- | --- | --- | --- |
| **Diagnosis** | **Peak age** | **Cumul. (%)** | **Peak age** | **Cumul. (%)** | **Peak age** | **Cumul. (%)** |
| **F600** | 24.10 | 0.43 | 23.00 | 0.37 | 24.85 | 0.55 |
| **F601** | 22.15 | 0.18 | 25.00 | 0.15 | 22.55 | 0.24 |
| **F602** | 23.10 | 0.24 | 30.95 | 0.19 | 30.10 | 0.29 |
| **F603** | 20.35 | 1.77 | 19.95 | 2.19 | 20.45 | 2.14 |
| **F604** | 29.85 | 0.04 | 22.45 | 0.04 | 29.60 | 0.06 |
| **F605** | 30.85 | 0.29 | 31.50 | 0.31 | 31.40 | 0.39 |
| **F606** | 23.00 | 1.84 | 22.20 | 2.21 | 23.80 | 2.50 |
| **F607** | 22.80 | 0.30 | 21.60 | 0.28 | 24.00 | 0.40 |
| **F608** | 28.10 | 0.18 | 32.10 | 0.12 | 28.85 | 0.23 |
| **F609** | 21.70 | 1.03 | 21.20 | 0.78 | 21.90 | 1.29 |
| **F61** | 22.95 | 1.18 | 22.30 | 1.19 | 27.10 | 1.52 |
| **Any** | 21.60 | 5.38 | 20.90 | 5.74 | 21.85 | 6.85 |

Supplementary table 1. Comparison of epidemiological estimates from the standard analysis and the two sensitivity approaches: long washout and no immigrants. Estimates from the three analyses generally converge.

*Supplementary figure 1*

Supplementary figure 1. **A.** Cumulative incidences across the lifespan for all four censoring states: death, emigration, any personality disorder diagnosis, and none of the above (survival), calculated in the entire sample. **B.** Cumulative incidences across the lifespan for all four censoring states calculated only among people born in Norway. Emigration constitutes a less dominant competing risk among people born in Norway.
